# Genome sequence of *Pectobacterium brasiliense* WZZ2024, isolated from fast-growing cabbage in China

**DOI:** 10.1128/mra.00770-25

**Published:** 2026-03-30

**Authors:** Zhen Zeng, Xiaorong Huang, Yin Jiang, Defeng Chen, Zunzheng Wei, Hao Liang

**Affiliations:** 1 National Engineering Research Center for Vegetables, Beijing Vegetable Research Center, Beijing Academy of Agriculture and Forestry Sciences 107624, Beijing, China; 2 Institute of Grassland, Flowers and Ecology, Beijing Academy of Agriculture and Forestry Sciences 107624, Beijing, China; 3 Key Laboratory of Urban Agriculture (North China), Ministry of Agriculture and Rural Affairs 12654 , Beijing, China; 4 College of Horticultural Science & Technology, Hebei Key Laboratory of Horticultural Germplasm Excavation and Innovative Utilization/Hebei Higher Institute Application Technology Research and Development Center of Horticultural Plant Biological Breeding, Hebei Normal University of Science and Technology 165079, Qinhuangdao, China; Wellesley College, Wellesley, Massachusetts, USA

**Keywords:** genome

## Abstract

The complete genome of *Pectobacterium brasiliense* WZZ2024, isolated from soft rot-infected petioles and tubers of fast-growing cabbage (*Brassica rapa* var. *glabra Regel*), comprises a single 4.83 Mb chromosome (GC content: 52.16%).

## ANNOUNCEMENT

*Pectobacterium brasiliense,* a widely distributed bacterial phytopathogen, induces soft rot disease in economically significant crops (e.g., potato, kiwifruit, and cabbage) by producing pectolytic enzymes that degrade plant cell walls (1). This species exhibits heightened pathogenicity compared to other *Pectobacterium* spp. (2), causing tissue maceration and substantial agricultural losses.

*P. brasiliense* WZZ2024 was isolated from soft rot-infected petioles and tubers of fast-growing cabbage, collected in the Vegetable Research Center of Beijing Academy of Agriculture and Forestry Sciences, China (August 2022). Tissue samples were surface-sterilized in 75% ethanol, rinsed with sterile distilled water (SDW), and homogenized aseptically. The resulting homogenates were plated onto Luria-Bertani Agar (LB agar) and incubated at 28°C for 12 h. Single colonies were isolated by successive streaking to achieve purity. Axenic cultures derived from these colonies were grown, and genomic DNA was extracted using the FastPure Bacteria DNA Isolation Mini Kit (Vazyme Biotech, China) according to the manufacturer’s instructions. The extracted DNA was subsequently subjected to whole-genome sequencing (3).

Library preparation and genome sequencing were performed at Biomarker Technologies (Beijing, China) on the PacBio Sequel II platform. Genomic DNA was sheared using g-TUBE (Covaris). The fragments were subjected to damage and end-repair using the SMRTbell Express Template Prep Kit 3.0 (PacBio, USA), followed by ligation of SMRTbell adapters and exonuclease digestion to remove unligated products. Size selection was performed using BluePippin (Sage Science, USA) to enrich fragments. Sequencing was conducted on the PacBio Sequel II platform using Sequel II Binding Kit 2.0 (PacBio, #101-842-900) and Sequel II Sequencing 2.0 Bundle (PacBio, #101-849-000) with HiFi chemistry v2.0. Post-processing steps retained high-quality CCS reads (>2 kb) using Filtlong v0.2.0 (--min_mean_q 20) for quality control. A total of 47,337 clean reads were obtained, with an average length of 9,475 bp. For Illumina sequencing, libraries were constructed and sequenced on the NovaSeq 6000 platform, yielding 3,292,417 read pairs, corresponding to 987.7 Mb of sequence data. Quality control was performed with Fastp v0.23.2 to remove adapters and low-quality reads. The Illumina data are available under SRA accession SRR33800100.

*De novo* assembly was performed using Unicycler v0.4.7 with both Illumina short reads and PacBio HiFi reads as input (4). Unicycler utilizes a multi-step hybrid approach: initial assembly graphs were constructed from the high-accuracy Illumina data using SPAdes v3.15.5, followed by bridging of contigs and gap-filling using the long PacBio reads. The assembly was subsequently polished with Pilon v1.22 (--mindepth 1 --changes --fix all) using available Illumina short-read data for error correction. Circularization and orientation were performed using Circlator v1.5.5 (minimus2 --no_pre_merge). Default parameters were used for all software unless explicitly stated. The final genome assembly of *P. brasiliense* WZZ2024 consists of a single circular chromosome of 4,831,335 bp with a G+C content of 52.16% (Table 1). The topology of the contig was predicted to be circular.

**TABLE 1:** Characteristics of *Pectobacterium brasiliense* WZZ2024

Characteristic	Result
Sequencing	
Raw reads	
No. of reads	47,456
Size (bp)	448,776,619
Clean reads	
No. of reads	47,337
Size (bp)	448,564,314
N_50_ (bp)	10,415
Average read length (bp)	9,475
Genome	
Assembly size (bp)	4,831,335
Contig	1
GC content (%)	52.16
No. of coding genes	4,212
Total gene size (bp)	4,178,577
Average gene length (bp)	992
Total repetitive sequence length (bp)	6,703
Repetitive sequence content (%)	0.14
rRNA	8, 7, 7 (5S, 16S, 23S)
tRNA	78
CRISPR	4
Genomic island	4
Prophage	4
Gene cluster	7
Protein subcellular localization	
Signal peptide	411
Transmembrane protein	1,038
Secreted protein	411

Public genome assemblies were annotated using NCBI PGAP v5.3 (5), with coding sequences predicted via Prodigal v2.6.3 (6). Repeat elements were identified by RepeatMasker v4.0.5 (7), while tRNA and rRNA genes were annotated using tRNAscan-SE v2.0 and Infernal v1.1.3, respectively (8–10). Functional features were predicted through: CRISPR arrays: CRT v1.2 (11); Genomic islands: IslandPath-DIMOB v0.2 (12); Prophages: PhiSpy v2.3 (13); Biosynthetic clusters: antiSMASH v5.0.0 (14). Secretory protein candidates were identified using SignalP after TMHMM-based transmembrane domain filtering (15, 16). Analysis results are listed in Table 1.

## Data Availability

This whole-genome shotgun project has been deposited in DDBJ/ENA/GenBank under the accession CP195137.1 and BioProject accession number PRJNA1271328. The raw reads have been submitted to the SRA under accession numbers SRR33800099 and SRR33800100. The version described in this paper is the first version.
